# Clinical manifestations of four patients diagnosed with early-onset sarcoidosis or sarcoid-like syndrome

**DOI:** 10.1186/1546-0096-12-S1-P293

**Published:** 2014-09-17

**Authors:** Sara Murias, Rosa Alcobendas, Agustin Remesal, Jesus Peralta, Susana Noval, Juan Ignacio Arostegui, Rebeca Rodriguez-Pena, Ana Mendez, Rosa Merino

**Affiliations:** 1 Pediatric Rheumatology, University Hospital La Paz, Madrid, Spain; 2 Pediatric Ophthalmology, University Hospital La Paz, Madrid, Spain; 3 Immunology, Hospital Clinic , Barcelona, Spain; 4 Immunology, General Pediatrics, University Hospital La Paz, Madrid, Spain; 5 TBC

## Introduction

Sarcoidosis is a rare multisystemic granulomatous disease. Pulmonary involvement is common in adults, but any organ can be affected.

## Objectives

To describe the main features of 4 pediatric patients diagnosed with early-onset sarcoidosis (EOS) or sarcoid-like syndrome.

## Methods

Medical charts were reviewed.

## Results

Four patients wer enrolled, 2 female (cases 1 and 2) and 2 male (cases 3 and 4). Their main characteristics are shown in table 1. The age at disease onset was 1.5, 10, 0.6 and 11 years respectively. The time until diagnosis ranged from 4 days to 8 years. The two cases with EOS (1 and 3) had been previously diagnosed as Juvenile Idiopathic Arthritis. Both started at a short age with the classical triad and carried a heterozygous gain-of-function *NOD2* mutation. Patient 2 seemed to be a late-onset sarcoidosis but persistent hypogammaglobulinemia and poor antibody production suggested a CVID, despite she did not suffer from recurrent infections. Other granulomatous lung diseases were dismissed in this case. Finally, diagnosis in patient 4 was made according to his clinical manifestations and the slight increase in ACE level.

**Table 1:** Main features of the four enrolled children

	Clinical data	Analytical data	ACE	Non-caseating granulomata	Diagnosis
1	Arthritis, rash, uveitis	Heterozygous p.R334Q	27.5	Skin	EOS
		Mutation at *NOD2* gene			

2	Splenomegaly,	Lymphopenia			CVID
	lymphadenopathy,	Thrombocytopenia			Sarcoid like syndrome
	interstitial/nodular	Hypogammaglobulinemia	92	Lung	
	lung disease	Poor antibody responses			

3	Arthritis, rash, uveitis				
		Heterozygous p.C495Y	19	Skin	Blau’s syndrome
		Mutation at *NOD2* gene		Synovial	
				Liver	

4	Right facial paralysis, parotid enlargement, uveitis, high fever		74.8		Heerfordt’s syndrome

ACE = angiotensin converting enzyme serum level (normal 10-50 IU/l)

CVID = common variable immunodeficiency

## Conclusion

Diagnosis of sarcoidosis in pediatric patients is often delayed because the disease is not suspected. Pulmonary involvement occurs less frequently in pediatric than in adults patients. This condition requires multidisciplinary management.

## Disclosure of interest

None declared.

